# Identification of Naturally Processed Epitope Region Using Artificial APC Expressing a Single HLA Class I Allotype and mRNA of HCMV pp65 Antigen Fragments

**DOI:** 10.3390/vaccines10050787

**Published:** 2022-05-16

**Authors:** Hong-Seon Pyo, Cheol-Hwa Hong, Haeyoun Choi, In-Cheol Baek, Tai-Gyu Kim

**Affiliations:** 1 Department of Microbiology, College of Medicine, Catholic University of Korea, Seoul 06591, Korea; ghdtjs3921@naver.com (H.-S.P.); hongch@catholic.ac.kr (C.-H.H.); chlgosl@catholic.ac.kr (H.C.); 2 Department of Biomedicine & Health Sciences, College of Medicine, Catholic University of Korea, Seoul 06591, Korea; 3 Catholic Hematopoietic Stem Cell Bank, College of Medicine, Catholic University of Korea, Seoul 06591, Korea; icbaek@catholic.ac.kr

**Keywords:** epitope identification method, HLA class I, artificial antigen-presenting cell, T cell epitope, transcriptionally active PCR, mRNA, human cytomegalovirus, pp65

## Abstract

Recently, long synthetic peptides or in silico-predicted epitope peptides have been used to identify T cell epitopes, but these approaches may not be suitable for investigating naturally processed epitopes. Here, mRNAs, including fragments or predicted epitope sequences of HCMV pp65 antigen, were generated by in vitro transcription following transcriptionally active PCR. Then, artificial antigen-presenting cells (aAPCs) expressing a single HLA allotype were transfected with mRNAs to identify epitopes in donors with T cell responses that recognize pp65 antigen restricted to HLA-A*02:01, -A*02:06, or -B*07:02. T cells restricted to a particular HLA allotype showed positive responses in some of the 10 fragment antigens. Among predicted epitopes within these positive fragments, three epitopes of HLA-A*02:01, -A*02:06, and -B*07:02 were confirmed. In addition, T cells expanded by anti-CD3 stimulation for two weeks could also be effectively used for the identification of these T cell epitopes, although there were individual differences. These results demonstrated that fragment antigens and epitopes can be rapidly generated using mRNA, and naturally processed antigenic regions can be detected using aAPCs without a T cell cloning procedure. This method will help to identify novel T cell epitopes for developing immunotherapy and vaccines against infectious diseases and cancer.

## 1. Introduction

HLA antigens, a cell surface glycoprotein, are characterized by a high degree of allelic polymorphism within the human population [1]. The HLA molecule has a biological function of MHC-restricted antigen presentation, which presents pathogen-derived peptides to CD8^+^ cytotoxic T lymphocytes and CD4^+^ T-helper cells [2]. Peptide-HLA complexes bind to clonally T cell receptors (TCRs) [3]. Generally, most HLA class I binding peptides recognized by CD8^+^ cytotoxic T cells are 8–10 residues in size [4].

So far, several approaches have been developed for mapping T cell epitope peptides [5]. Peptide loading technologies (peptide libraries and MHC-peptide exchange technologies [6]), mass spectrometry analysis of MHC binding peptides for immunopeptidomics [7,8], and cDNA libraries screening [9] have successfully identified many T-cell epitopes in different diseases. However, these are often time-consuming and may sometimes miss the real epitope recognized by specific T cells [10]. In addition, peptide loading approaches do not consider alternative translational and post-translational structurally altered epitopes, and unrelated latent epitopes that are not processed by antigen-presenting cells (APCs) in vivo may be identified [11].

Alternatively, mRNA fragments were used to reveal the specificity of T cells, and transfection of DCs with mRNA was superior to other antigen-loading techniques [12]. In our previous studies, mRNA of several viral antigens, such as HCMV pp65 [13], EBV LMP1, LMP2a [14], and tumor antigens [15], were transferred to human dendritic cells to measure T cell immune response or to produce T cells in vitro for adoptive T cell therapy [16]. This technique uses plasmid DNA templates cleaved at restriction sites or new sites inserted by mutagenesis and generated mRNA fragments of varying lengths [17]. Transcriptionally active PCR (TAP) technology, which allows the direct use of linear DNA fragments, was used to individually screen each gene for immunogenic potential and developed in vitro expression, functional studies, and in vivo immunization [18]. Furthermore, a technique for producing antigenic mRNA by in vitro transcription (IVT) following TAP has been previously developed to rapidly and efficiently identify HLA class I or II epitopes recognized by antigen-specific CD8^+^ or CD4^+^ T lymphocytes [19].

Cytomegalovirus (CMV), a common herpes virus, is a major opportunistic pathogen that is frequently reactivated from latency during prolonged immunosuppression [20]. Despite the effective prevention of CMV disease with allogeneic T-cell-deficient stem cell transplantation, transplant-related morbidity and mortality are increased in CMV seropositive patients [21]. CTL epitopes of CMV are useful for monitoring individual antiviral immunity and for in vitro generation of antiviral CTLs for adoptive immunotherapy [22]. Lower matrix protein 65 (pp65), a structural protein abundant throughout CMV infection, is widely recognized as an immune-dominant target of the CD8^+^ T cell response to CMV [23].

Artificial antigen-presenting cells (aAPCs) are generally designed to express both HLA allotypes and important co-stimulatory molecules. It can present immunogenic viral or tumor antigens on a single expressed HLA allotype to generate HLA-restricted T cells of the desired specificity [24]. HLA-transfected K562 cells as aAPCs efficiently expressed whole antigens and presented antigenic peptides after electroporation with pp65 mRNA and different 3’-deleted pp65 mRNA fragments, but were limited to only one HLA class I allotype [17]. We previously established a panel of aAPCs expressing a single HLA class I or class II allotype [25] and performed comprehensive analyses for the pp65-specific CD8^+^ [26] and CD4^+^ T cell [27] responses restricted by a single HLA allotype within an individual.

Here, an approach was developed to rapidly identify fragments and epitopes of the pp65 antigen using mRNA. And in vivo-relevant epitopes restricted to a specific HLA class I allotype and recognized by previously defined antigen-specific CD8^+^ T cells could be efficiently identified using aAPCs. This method can be used to identify new T cell epitopes for the development of immunotherapy and vaccine for infectious diseases and cancers.

## 2. Materials and Methods

### 2.1. PBMCs and Ethics Statement

In a previous study, T cell responses to the CMV pp65 antigen were measured in 50 healthy volunteers using aAPC expressing a single HLA allotype. Among them, 9 donors with strong T cell immune responses were selected for this study and showed T cell responses restricted to -A*0201, -A*0206, and -B*0702, respectively [26]. Their HLA genotypes are presented in Supplementary Table S1. Their peripheral blood mononuclear cells (PBMCs) were obtained from the Catholic Hematopoietic Stem Cell Bank and cryopreserved 1 × 10^7^ in 40% RPMI 1640 medium (Lonza, Walkersville, MD, USA) supplemented with 50% fetal bovine serum (Gibco, Waltham, MA, USA) and 10% Cryserv (dimethyl sulfoxide, USP) (Mylan, Canonsburg, PA, USA). This study obtained written consent from all participants and approval from the Institutional Review Board (IRB) of the Catholic University of Korea (IRB number: MC22SASI0008).

### 2.2. In Vitro Expansion of T Cells

T cells from PBMCs were expanded for 2 weeks to obtain large amounts of T cells to replace limited number of T cells within PBMCs as effector cells. In a previous study, we used the CRISPR/CAS-9 method to create HEK293T cells (CRL-3216; ATCC, Manassas, VA, USA) in which all HLA Class I genes were removed, and then established artificial antigen-presenting cell (Null-293T(H1E-45)-Cos) expressing adhesion and costimulatory molecules (CD54, CD70, CD80, CD83, CD137L) and CD32 molecule to coat antibodies [25]. This cell line was used to non-specifically proliferate T cells using an anti-CD3 antibody or to measure HLA restricted T cell responses by transferring a single HLA allotype gene. PBMCs were cultured in RPMI 1640 medium (Lonza, Walkersville, MD, USA) supplemented with 10% fetal bovine serum (Gibco, NY, USA), 1% L-glutamine (Lonza, Walkersville, MD, USA), and 1% penicillin-streptomycin (Lonza, Walkersville, MD, USA). On day 0, 1 × 10^7^ PBMCs were stimulated with 100Gy irradiated aAPCs at a 10:1 T:aAPC ratio, 0.5 µg/mL OKT3 (eBioscienceTM, San Diego, CA, USA), and 20U/mL IL-2 (Clinigen Healthcare Ltd., London, UK) and seeded at 1 × 10^6^/well in 24 well plates. aAPCs and OKT3 were additionally stimulated under the same condition on day 7, and IL-2 was treated every 3–4 days. When the cells increased to 1 × 10^8^ or more during expansion, they were cultured in T75 flasks. On day 14, 1 × 10^7^ aliquoted cells were cryopreserved in liquid nitrogen.

### 2.3. Transcriptionally Active PCR (TAP) for pp65 Antigen

The primers of fragment antigens and predicted epitopes were designed with nucleic acid sequences on HCMV pp65 and HIV Gag obtained from NCBI (https://www.ncbi.nlm.nih.gov/gene/ accessed on 1 April 2021) (Supplementary Table S2). Since HIV becomes a permanent chronic infection after primary infection, T cells in healthy adults who are HIV-negative means that they are not sensitized to the HIV antigen. Therefore, many other studies measuring human T cell responses use HIV peptide as a negative control. In silico epitope prediction for each HLA restricted specific pp65 CD8^+^ T cell was obtained from the IEDB (https://www.iedb.org/ accessed on 10 April 2021): T cell Epitope Prediction-MHC I Binding; NetMHCpan EL 4.1 Prediction Method; human; HLA-A*02:01, -A*02:06, or -B*07:02; All lengths; Predicted Score (descend). To identify epitopes with high binding affinity, only epitopes with a prediction score of 0.7 or higher were aligned.

Ten fragments with 70 amino acids (aa) of pp65 antigen were amplified using 35 base pair (bp) forward and reverse primers so that each 15aa overlapped. The T7 promoter-Kozak sequence to be used for TAP was amplified from the T7 promoter vector. TagBFP was amplified so that T2A-TagBFP-Beta globin pA was sequentially linked. Afterward, the TAP products were sequentially linked. The 15aa mini-genes, so that the 8–12aa epitope sequence predicted from IEDB was centered, were amplified. For example, NLV, a predicted epitope located at 495–503aa, amplified the 492–506aa sequence. The subsequent process was performed with the same design as the fragment antigens. All primers were ordered from Cosmo Genetech (Seoul, Korea) and TAP proceeded as follows according to the PCR cycle conditions of the KOD FX kit (Toyobo, Osaka, Japan): 2 min at 98 °C in one cycle, 10 s at 98 °C, 30 s at 60 °C, 1 min at 68 °C in 34 cycles, and 5 min at 68 °C in one cycle. In the annealing step, it was performed for 1 min at 1 kb and increased by 30 s in units of 500 bp according to the predicted gene size. After the concentration of the completed TAP product was confirmed with nanodrop, the diluted TAP products were electrophoresed on 2% agarose gel using SYBR Green with a 100 bp or 1 kb ladder (Bioneer, Daejeon, Korea) at 200 V for 20 min. For the TAP products matching the predicted gene size, only the templates were neatly separated according to the manual of the PCR clean-up kit (MN, Dueren, Germany). The completed templates were stored at −4 °C.

### 2.4. In Vitro Transcription of pp65 Antigen mRNA from TAP Products

Using 500 ng of TAP products, mRNA in vitro transcription was performed overnight according to the manual of the MEGAscript T7TM Kit (Invitrogen, Vilnius, Lithuania), and polyadenylation at the 3’-terminals was performed according to the manual of the poly (A) Tailing Kit (Invitrogen, Vilnius, Lithuania) to increase the efficiency of translation initiation. Complete mRNA was purified according to the manual of the MEGAclear™ Kit (Invitrogen, Vilnius, Lithuania). After confirming the concentration of mRNA with nanodrop, it was aliquoted at an appropriate concentration for safe use and stored at −80 °C. The RNA was used within at least three weeks because the efficiency weakens over time. All places touched by hands were wiped with RNaseZap (Invitrogen, Vilnius, Lithuania), and the experiment was carried out while maintaining a cool temperature.

### 2.5. Electroporation of mRNA to aAPCs Expressing a Single HLA Class I Allotype

HEK293T cell line expressing each HLA-A*0201, -A*0206, –B*0702 allotype were cultured in RPMI 1640 medium (Lonza, Walkersville, MD, USA) supplemented with 10% fetal bovine serum (Gibco, NY, USA), 1% L-glutamine (Lonza, Walkersville, MD, USA) and 1% penicillin-streptomycin (Lonza, Walkersville, MD, USA). Adherent cells were treated with 0.25% Trypsin-EDTA (Gibco, Waltham, MA, USA) and washed twice with optiMEM (Gibco, Waltham, MA, USA). Hence, 1 × 10^6^ aAPCs and 60 µg mRNA expressing whole antigen, fragments, or predicted epitopes of pp65 were added in a 2 mm gap cuvette and transfected using a Square Wave Electroporation System (BTX, New York, NY, USA) at 400 V, 500 µs. Immediately after electroporation, the cells were washed with cultured media and incubated at 37 °C for 24 h. After that, transfected aAPCs were analyzed for BFP expression by flow cytometry and used to stimulate CD8^+^ T cells.

### 2.6. IFN-γ ELISA

In each well, 5 × 10^5^/100 µL T cells and 5 × 10^4^/100 µL transfected aAPCs were co-cultured in 96 well plates at a 10:1 ratio and incubated at 37 °C for 24 h. After that, the supernatant was separated from the co-cultured cells by centrifugation and stored at −4 °C. The collected supernatant was then subjected to cytokine analysis according to the manual of the Human IFN-γ ELISA kit (Invitrogen, Graz, Austria) in 96-well U-bottom plates. Briefly, the ELISA plate was coated with anti-human IFN gamma antibody (capture antibody) overnight at 4 °C, washed 3 times, and blocked with 200 µL of 1X Assay Diluent A for 1 h at room temperature (RT). Then, after one wash, the standard and the collected supernatant (100 µL/well) were added and incubated at 4 °C overnight, after four washes, and incubated with biotin-conjugated anti-human IFN gamma antibody (detection antibody) for 1 h at RT. After 4 washes, incubated with Streptavidin-HRP for 30 min at RT, after 6 washes, and incubated with 1X TMB Solution for 15 min at RT in the dark. The reaction was stopped with Stop Solution (Invitrogen, Graz, Austria). Antibody levels were measured at 450 nm in a Synergy H1 microplate reader (BioTek, Winooski, VT, USA). The lowest among the values for each fragment antigen or predicted epitope was used as a background value and subtracted from all values.

### 2.7. Flow Cytometry

HLA and co-stimulatory molecules of aAPCs, BFP expression of mRNA-transfected aAPC, and proportion of PBMCs and expanded T cells were analyzed by flow cytometry (FACS Canto, BD, Franklin Lakes, USA). Cells were stained with the following antibodies: APC Mouse anti-human HLA-ABC (G46-2.6(RUO), BD, Franklin Lakes, NJ, USA), PacificBlueTM anti-human CD54 (HCD54, Bio Legend, San Diego, CA, USA), APC anti-human CD32 (FUN-2, Bio Legend, San Diego, CA, USA), FITC anti-human CD70 (113-16, Bio Legend), Anti-human CD80 PE-Cy5 (2D10.4, e-Bioscience, Frankfurt, Germany), Anti-human CD83 (HB15e, BD), PE anti-human 4-1BB Ligand(CD137L) (5F4, Bio Legend), Brilliant Violet V510 anti-human CD56(NGAM) (1H11, Bio Legend), Brilliant Violet 421 anti-human CD3 (OKT3, Bio Legend), APC anti-human CD4 (RPA-T4, Bio Legend), and PE anti-human CD8a (HTT8a, Bio Legend).

### 2.8. Statistical Analysis

FlowJo v10 (BD) and Prism 5.01 software (GraphPad Software, San Diego, CA, USA) were used for data analysis and visualization. Statistical significance was determined by paired *t*-test, one-way ANOVA, Dunnett’s multiple comparison test, and Pearson’s correlation analysis. Graphs are shown as means ± standard deviation (SD). Values of *p* < 0.05 were considered significant.

## 3. Results

### 3.1. Generation of mRNA including HCMV pp65 Antigens

It is the purpose of this study to establish a method for efficiently identifying epitopes. This allows aAPCs expressing a single HLA class I allotype to naturally present as the form of endogenous antigen. First, the epitope region was identified by transferring mRNA containing fragments overlapping 15aa covering all antigenic sites to aAPCs and examining the T cell response. Then, the predicted epitopes in the fragments where the reaction was positive were sequentially identified in the same way to finally confirm the epitopes.

**Figure 1:**
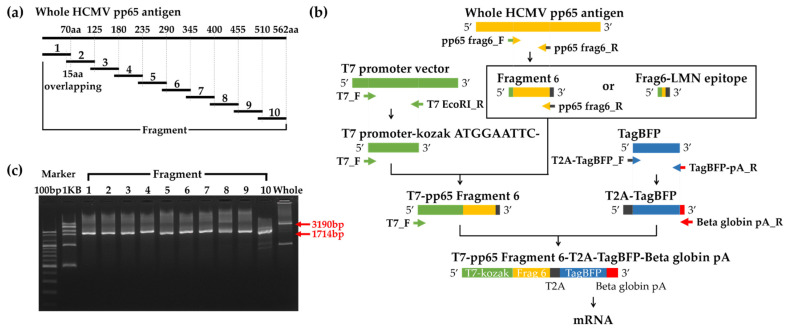
Generation of mRNA including pp65 whole and fragment antigens by TAP. (**a**) Ten fragments of the pp65 antigen consist of 70aa and are overlapped by 15aa. (**b**) Schematic summary of TAP process. Fragment 6 and the LMN epitope are exemplified. Finally, the T7 promoter-Kozak-pp65 whole or fragment or predicted epitope-T2A-BFP-beta-globin pA was amplified by TAP. mRNA for electroporation was produced by IVT of TAP products. (**c**) 2% Agarose gel electrophoresis of DNA amplified by TAP. The band size of the whole and fragment TAP products are 3190bp and 1714bp, respectively.

The pp65 antigen was selected as an antigen suitable for this study because the frequency of T cells that respond strongly even in healthy persons is high due to its high antigenicity and the epitope is well defined. The pp65 antigen was divided into ten fragments with a length of about 70aa, and 15aa overlapped between each fragment (Figure 1a). To generate a TAP product to express the antigen, the following regions were amplified by PCR: the antigenic region containing each pp65 whole or fragment, the transcription region containing the T7 promoter-Kozak sequence, and the fluorescent region containing the T2A, BFP, and beta globlin poly-A sequences. These PCR products with 15bp overlap on both sides were linked sequentially by overlapping PCR (Figure 1b). Each TAP product showed the predicted size by agarose electrophoresis (Figure 1c).

### 3.2. Artificial Antigen-Presenting Cells Expressing a Single HLA Class I Allotype and pp65 Antigen

**Figure 2:**
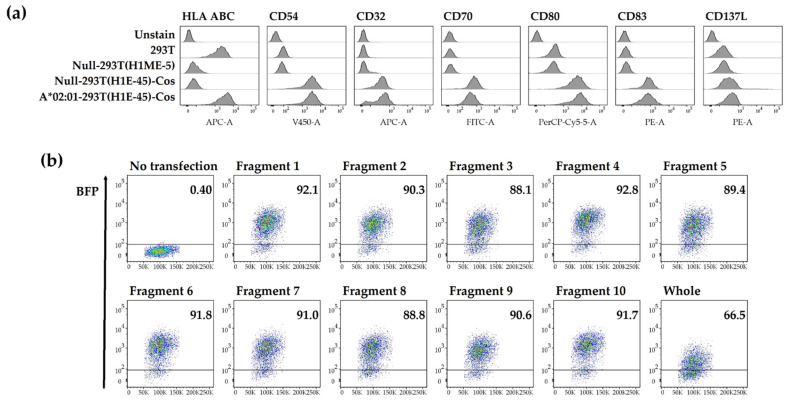
Transfer of mRNA including pp65 whole and fragment antigens to aAPCs expressing a single HLA class I allotype. (**a**) HLA and co-stimulatory molecules (CD54, CD32, CD70, CD80, CD83 and CD137L) expression in the HEK293T cell line were measured by flow cytometry. A*02:01-293T(H1E-45)-Cos is to express HLA-A*02:01 in Null-293T(H1E-45)-Cos. (**b**) The expression of BFP co-expressing pp65 antigen in A*02:01-293T(H1E-45)-Cos cells were measured by flow cytometry.

Null-293T(H1E-45)-Cos cells established as aAPCs in a previous study enabled restricted antigen presentation to a single HLA class I allotype [25]. These cells transduced a co-stimulatory molecule into Null-293T (H1ME-5) cells which HLA class I was knocked out from HEK293T cells and then transduced with lentivirus vectors expressing a single HLA class I allotype, such as HLA-A*02:01, -A*02:06, or -B*07:02. The expression of a single HLA class I allotype and co-stimulatory molecules was analyzed by flow cytometry (Figure 2a). The expression of HLA-A*02:01 is representatively shown in Figure 2a, and also confirmed in A*02:06-293T (H1E-45)-Cos cells and B*07:02-293T (H1E-45)-Cos cells.

To confirm the expression of the antigen in A*0201-293T(H1ME-5)-Cos, the expression of BFP linked to pp65 fragment antigens by T2A was analyzed by flow cytometry. BFP expression was over 88% in cells transfected with fragments but was 66% in cells transfected with the whole antigen. This could be due to the difference in molar concentration caused by the delivery of the same amount of mRNA even if the antigens are of different sizes (Figure 2b). BFP expression was confirmed similarly in aAPCs expressing other HLA class I allotypes and used to measure T cell responses.

### 3.3. In Vitro Expansion of T Cells to Identify Epitopes

**Figure 3:**
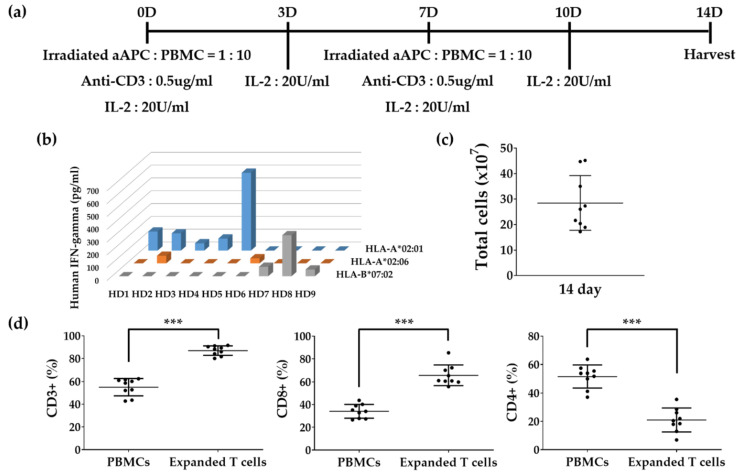
In vitro expansion of T cells. (**a**) Overview of T cell expansion from PBMCs using aAPCs expressing co-stimulatory molecules and anti-CD3. (**b**) HLA restriction and reactivity of 9 donors with positive T cell response to pp65 antigen. PBMCs of each donor were cultured with aAPCs expressing a single HLA class I allotype and pp65 whole antigen for 24 h and the level of IFN-γ in the culture supernatants was measured by ELISA. (**c**) Total number of cells at 14 day after the expansion of PBMCs. Dots represent individual donors (n = 9). Error bars show the median ± in-terquartile range. (**d**) Changes in the proportion of CD3^+^, CD8^+^, and CD4^+^ T cells after two weeks of expansion were measured by flow cytometry. Dots represent individual donors (n = 9). *p* value was calculated by Paired *t*-test. *** *p* < 0.0001.

**Figure 4:**
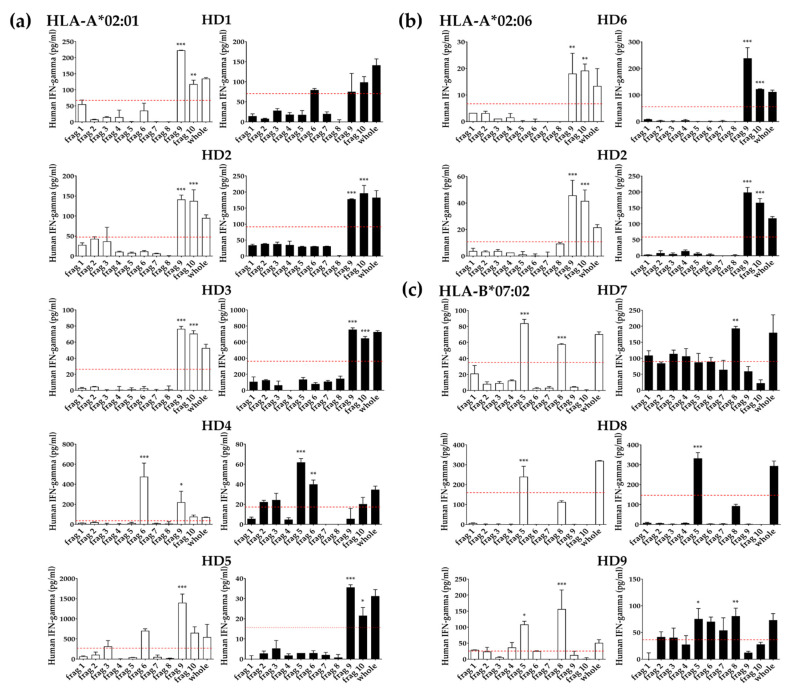
Identification of epitope regions using aAPCs expressing pp65 fragment antigens. PBMCs (white bar) and expanded T cells (black bar) of each donor were co-cultured with aAPCs expressing a single HLA class I allotype such as (**a**) HLA-A*02:01, (**b**) HLA-A*02:06, and (**c**) HLA-B*07:02 and pp65 whole or fragment antigens at a ratio of 10:1 for 24 h and the level of IFN-γ in the culture supernatants was measured by ELISA. Red dotted line; positive cut-off value. All experiments were performed in triplicate and repeated at least twice. Representative data are shown. Error bars show mean values ± SD of triplicate. *p* value was calculated by one-way ANOVA. *; *p* < 0.05, **; *p* < 0.005, ***; *p* < 0.0005.

Since the amount of blood collected from volunteers is limited to perform repeated trials to define multiple fragment antigens or predicted epitopes, we expanded T cells for two weeks. ELISA tests using PBMCs and expanded T cells were performed simultaneously (Figure 3 and Figure 4). Compared with the number of cells on 0 day of 0.7 × 10^7^ ± 0.2 × 10^7^ (average ± SD), the number of cells on day 14 increased to 28.5 × 10^7^ ± 10.7 × 10^7^ (Figure 3c). The proportion of CD3^+^ T cells (1.6 ± 0.2) and CD8^+^ T cells (2.0 ± 0.3) in expanded T cells increased compared to PBMCs. However, the proportion of CD4^+^ T cells (3.0 ± 1.9) in expanded T cells decreased compared to PBMCs.

Five HLA-A*02:01 positive donors (HD1, HD2, HD3, HD4, and HD5), two -A*02:06 positive donors (HD2 and HD6), and three -B*07:02 positive donors (HD7, HD8, and HD9) who were found to have CD8^+^ T cells specific to pp65, which is restricted by a particular HLA class I allotype in a previous study, were selected. The HD2 donors showed CD8^+^ T cell responses in both HLA-A*02:01 and -A*02:06 [26]. The T cell response specific to pp65 whole antigen restricted in the selected PBMCs was confirmed by IFN-γ ELISA using aAPCs expressing these HLA allotypes (Figure 3b). All PBMCs expressed various IFN-γ values and were expanded by the above expansion method. There was little difference in the freeze and thaw viability of expanded T cells (82.2 ± 14.5).

### 3.4. Identification of T Cell Epitope Region Using aAPCs

**Figure 5:**
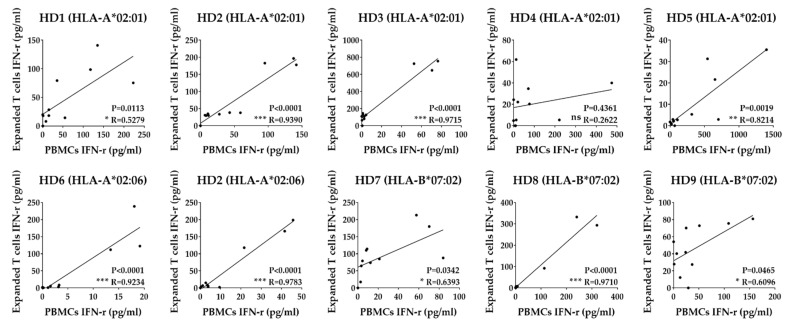
Correlation between T cell response to pp65 antigens by PBMCs and expanded T cells. Dots represent IFN-γ values secreted by PBMCs and expanded T cells specific for individual pp65 whole and fragment antigens. *p* value by two-tailed *t*-test and R value was calculated by Pearson’s correlation analysis. ns; *p* > 0.1, *; *p* < 0.05, **; *p* < 0.01, *** *p* < 0.001.

Since the T cells used in this study have already been defined to recognize the pp65 antigen presented by a specific HLA class I allotype using aAPCs, we can conversely use this T cell to further identify fragments, including the epitope of pp65 or the epitope itself. To identify the epitope regions of the pp65 antigen, PBMCs and expanded T cells were stimulated with aAPCs expressing a single HLA class I allotype and expressing each pp65 fragment antigen. It was observed which fragment antigen showed a positive reaction in each HLA by IFN-γ ELISA (Figure 4). Fragments with values significantly higher than half of the whole antigen value were defined as a positive response. We also analyzed the correlation of T cell response between PBMCs and expanded T cells in each donor (Figure 5). All donors except for the HD4 donor showed a significant correlation. In particular, HD2, HD3, HD5, HD6, and HD8 donors showed a strong correlation (*p* < 0.001).

Among donors responding to HLA-A*02:01, HD2 and HD3 donors showed positive responses in fragments 9 and 10 by PBMCs and expanded T cells (Figure 4a). HD1 donor showed positive responses in fragments 9 and 10 only in PBMCs. The HD5 donor showed positive responses in fragments 9 and 10 by expanded T cells and fragment 9 by PBMCs. The HD4 donor specifically showed positive responses in fragments 6 and 9 by PBMCs, and fragments 5 and 6 by expanded T cells. HD2 and HD6 donors responding to HLA-A*02:06 showed positive responses in fragments 9 and 10 by PBMCs and expanded T cells (Figure 4b). Among donors responding to HLA-B*07:02, the HD7 donor showed positive responses in fragments 5 and 8 by PBMCs, and only in fragment 8 by expanded T cells (Figure 4c). The HD8 donor showed a positive response in fragment 5 by PBMCs and expanded T cells. Fragment 8 of HD8 was not a significant positive response by the defined positive cut-off value, but it was considered a positive response based on the background value. The HD9 donor showed positive responses in fragments 5 and 8 by PBMCs and expanded T cells.

### 3.5. Identification of Epitopes within Fragment Antigens

**Figure 6:**
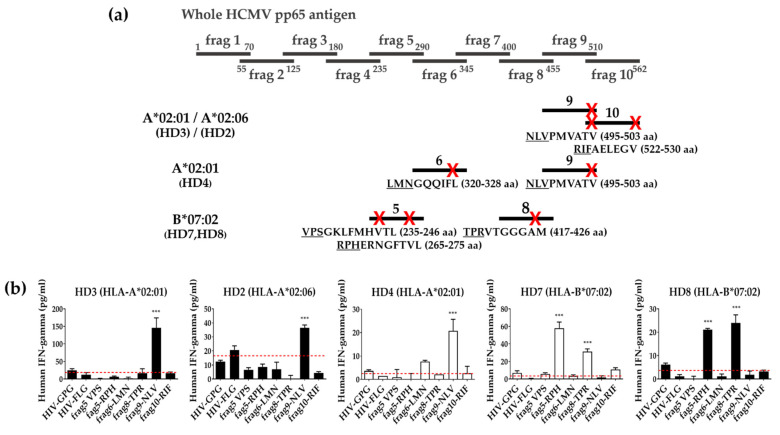
Identification of T cell epitopes within fragment antigens using aAPCs expressing pp65 predicted epitopes. (**a**) Summary of donors, pp65 fragment antigens, and predicted epitopes with T cell response restricted to specific HLA class I allotype. Epitopes with a prediction score of 0.7 or higher in the pp65 positive fragments marked a red X at that region and written below it. (**b**) PBMCs(white bar) or expanded T cells(black bar) of each donor were co-cultured with aAPCs expressing a single HLA class I allotype such as HLA-A*02:01, -A*02:06, or -B*07:02 and mini-genes containing each predicted epitope at a ratio of 10:1 for 24 h and the level of IFN-γ in the culture supernatants was measured by ELISA. HIV epitopes were used as negative controls. HIV-GPG; HLA-B*07:02 restricted epitope within Gag antigen, HIV-FLG; HLA-A*02:01 restricted epitope within Gag antigen. Red dotted line; positive cut-off value. All experiments were performed in triplicate. The mean values ± SD of triplicate. *p* value was calculated by one-way ANOVA. ***; *p* < 0.0005.

For more detailed epitope identification, donors who tested positive for the same fragments were grouped. Six mini-genes with a length of 15aa containing the epitope having a predicted score of 0.7 or higher in the pp65 positive fragment antigens were amplified in the same way as the fragments (Figure 6a). IFN-γ ELISA of PBMCs or expanded T cells was performed using aAPCs expressing predicted epitopes and a single HLA class I allotype (Figure 6b). Predicted epitopes with values significantly higher than half of the average value of the two epitopes of the HIV Gag peptide were defined as a positive response.

When three candidate epitopes in fragments 9 and 10 were evaluated in the HD3 donor of HLA-A*0201 and HD2 donor of -A*0206, the NLV epitope located overlappingly in fragments 9 and 10 showed positive responses, but the RIF epitope of fragment 10 did not show any positive response. In the HD4 donor showing positive responses in fragments 6 and 9 by PBMCs, the NLV epitope located in both fragments 9 and 10 showed a positive response, but the LMN epitope of fragment 6 did not show any positive response. When three candidate epitopes in fragments 5 and 8 were evaluated in HD7 and HD8 donors of HLA-B*0702, the RPH epitope of fragment 5 and the TPR epitope of fragment 8 showed positive responses, but the VPS epitope of fragment 5 did not show any positive response.

## 4. Discussion

In this study, we tried to develop a method using TAP and IVT mRNA to facilitate the identification of novel epitopes of antigens (Figure 1). The synthesized peptides have immunogenicity to induce specific T cell responses and high binding affinity with relevant MHC molecules in vitro [28]. Even with an accurate prediction of potential peptide candidates in silico, the number of epitopes ignored in screening is significant. Recently, long synthetic peptides that overlap each other at the protein level have been used for the entire length of the antigen [29]. However, these peptides were not generated through natural processing and presentation steps. In another attempt, by generating easily linear DNA containing the promoter and antigen genes through TAP, cloning for the expression of various genes was effective, and immunogenicity among many antigens in infectious agents could be screened [30]. However, we found that the direct delivery of these long TAP products with low in vitro production to APCs was inefficient. In this study, mRNA amplified about 100 times was generated through IVT. In addition, the condition for mRNA transfer using electroporation had a higher survival rate and was appropriate for T cell stimulation because it caused less damage to cells than DNA transfer [31].

Generally, to identify the epitopes recognized by T cells, it is necessary to simultaneously examine which epitope is presented by which HLA allotype by using autologous or partially matching APCs. In our previous study, using K562-based aAPCs expressing a single HLA, it was possible to determine which HLA class I allotype pp65 antigen-specific CD8^+^ T cells are presented. In particular, pp65 antigen-specific CD8^+^ T cells in peripheral blood recognized antigens presented by one or both allotypes despite expression of up to six HLA class I allotypes in one individual [26]. We referred to the T cell response to a small number of HLA allotypes in one individual as allele dominance. Therefore, it was confirmed that the T cells used in this study did not show a response to other HLA allotypes. An average of 10% of CD8^+^ memory T cells in the T cell response to HCMV are directed against the virus [32]. In addition, immune responses to HCMV peptides in a latent-infected individual are characterized by a few highly concentrated, massively expanded CD8^+^ T cell clones [33]. Therefore, in this study, eight donors whose responded to a single HLA-A*02:01, -A*02:06, or -B*07:02 allotype with pp65 antigen-specific CD8^+^ T cells and 1 donor (HD2) responded to both -A*02:01 and -A*02:06 were used for epitope identification. Additionally, we previously generated Null-293T(H1E-45)-Cos cells that do not express HLA class I using CRISPR/CAS-9 to improve K562-based aAPCs [25]. The novel aAPCs system using these cells facilitated gene transfer, had a low non-specific T cell response, and enabled the expression of various co-stimulatory molecules. Thus, it stimulates T cells efficiently and enables antigen-specific T cell proliferation in vitro.

In a comprehensive study of CD8^+^ T cell responses specific for HCMV, EBV, HIV, and HCV [34], virus-specific CD8^+^ cells underwent a linear differentiation program as follows. CD45RA^+^ CD28^+^ CD27^+^ CCR7^+^ naive cells became CD45RO^+^ after activation and the expression of CCR7, CD28, and CD27 sequentially lost. The predominant phenotype for each virus accumulated at different points along the pathway, and HCMV-specific cells were predominantly a late differentiation phenotype when CD45RA was re-expressed [35]. Based on a previous report that antigen-non-specific in vitro expansion of T cells does not affect the repertoire of TCRs [36], we performed in vitro expansion of T cells to obtain many T cells required for antigen screening. By culturing for two weeks, the total number of cells increased by an average of 40 times, and the ratio of T cells and CD8^+^ T cells increased, but CD4^+^ T cells decreased (Figure 3c). The responsiveness of CD8^+^ T cells specific to pp65 fragments in PBMCs and expanded T cells showed a significant correlation except for the HD4 donor (Figure 5). The CD8^+^ T cell response to each pp65 fragment showed a similar pattern, but in some cases, they were inconsistent. In particular, the intensity of pp65-specific CD8^+^ T cell response in expanded T cells from HD3 and HD6 donors was increased by about 10 times compared to PBMCs, whereas HD4 and HD5 decreased by more than 10 times (Figure 4). These results suggest that the degree of differentiation of pp65-specific CD8^+^ T cell clones in PBMCs varies from individual to individual. Therefore, PBMCs and expanded T cells can be used complementarily to identify T cell epitopes.

For a comprehensive evaluation of the antigen’s CD8^+^ T cell epitope, 70aa fragments overlapping 15aa were used primarily, and the predicted epitopes within the fragments then investigated showed a positive response in the same way. In screening with pp65 fragment antigens, Fragments 6, 9, and 10 were positive in HLA-A*02:01 positive donors, and fragments 9 and 10 were positive in -A*02:06 positive donors. Here, it was assumed that both HLA-A*02:01 and -A*02:06 would have an epitope region in the overlapping region of the two fragments based on the continuous reaction of fragments 9 and 10. As a result of measuring the response to epitopes with a high prediction rate (>0.7) among predicted epitopes within the fragments showing a positive response, the NLV epitope located at the overlapping site of fragments 9 and 10 showed a positive reaction (Figure 6). In addition, based on the positive reaction of fragments 5 and 8 in the HLA-B*07:02 donor, it was speculated that there were two types of clones. Moreover, the epitope in fragments 5 and 8 also showed a positive reaction, respectively, suggesting the existence of two clones. These speculations can further confirm a more accurate response using a single TCR or cloned T cell [33]. However, the HD4 donor showing a positive reaction to fragment 6 presented by HLA-A*0201 did not show a clear positive reaction to the predicted epitope within fragment 6, but only a weak reaction. Peptide binding is uncompromising to frameshifts of anchor residues within the peptide sequence because of the closed ends of the peptide-binding grooves [37]. Therefore, to screen without missing epitopes, it may be necessary to confirm using shorter fragments that completely cover the fragment showing a positive response.

## 5. Conclusions

In summary, it was demonstrated with the pp65 antigen that antigenic epitopes recognized by T cells responding to a particular antigen can be rapidly identified using aAPC expressing a single HLA allotype and fragment antigen mRNA generated through TAP. By measuring the response to a single HLA allotype, it will be possible to facilitate epitope identification for complex T cell clones in peripheral blood without T cell cloning. Moreover, it was suggested that using non-specifically expanded T cells has the advantage of analyzing epitopes recognized by memory T cells with low frequency in addition to securing a large number of cells.

## Data Availability

The data presented in this study are available within the article.

## Supplementary Materials

The following supporting information can be downloaded at: https://www.mdpi.com/article/10.3390/vaccines10050787/s1, Supplementary Table S1. Donors HLA genotypes. Supplementary Table S2. PCR primer sequence for DNA template TAP.
