# Supplementary material for: Identification of Naturally Processed Epitope Region Using Artificial APC Expressing a Single HLA Class I Allotype and mRNA of HCMV pp65 Antigen Fragments
Source: Vaccines (Basel). 2022 May 16;10(5):787. doi: 10.3390/vaccines10050787 (PMC9143612; doi:10.3390/vaccines10050787)
Supplement: Supplementary file 1 [file vaccines-10-00787-s001.zip › vaccines-1686404-supplementary.pdf]

vaccines MDPI

**Supplementary Table S1.** Donors HLA genotypes.

| Donor | A1 | A2 | B1 | B2 | C1 | C2 |
|---|---|---|---|---|---|---|
| HD1 | 0201 | 3303 | 3501 | 4403 | 0401 | 1403 |
| HD2 | 0201 | 0206 | 1501 | 1511 | 0303 | 0702 |
| HD3 | 0201 | 1101 | 1501 | 4001 | 0304 | 0401 |
| HD4 | 0201 | 1101 | 1501 | | 0401 | 0801 |
| HD5 | 0201 | | 4801 | 5401 | 0304 | 0801 |
| HD6 | 3101 | 0206 | 1501 | 4601 | 0102 | 0303 |
| HD7 | 0201 | 2402 | 0702 | 5101 | 0702 | 1402 |
| HD8 | 0201 | 2402 | 0702 | 1501 | 0102 | 0702 |
| HD9 | 2402 | 3303 | 0702 | 3501 | 0702 | 0801 |

*Allotypes presenting the pp65 antigen are marked in red.

**Supplementary Table S2.** PCR primer sequence for DNA template TAP.

| Primer | Sequence(5' - 3) | Length(bp) |
|---|---|---|
| CMVT7_F | GTTGACATTGATTATTGACT | 20 |
| CMVT7_R | GAATTCCATGGTGGCAAGCTTGGGTC | 26 |
| T2A-TagBFP_F | GAGAATCCCGGCCCTATGTCCGAGCTGATTAAGGA | 35 |
| T2A-TagBFP_R | AGTCAGATGCTCAAGTCAATTAAGCTTGTGCCCCA | 35 |
| Beta globin pA_R | ACAAAAAATTCCAACACACTATTGCAATGAAAA-TAAATTTCCTTTATTAGCCAGAAGTCAGATGCTCAAG | 70 |
| pp65 whole_F | GCCACCATGGAATTCATGGAGTCGCGCGGTCGCCG | 35 |
| pp65 whole_R | ACTTCCTCTGCCCTCACCTCGGTGCTTTTTGGGCG | 35 |
| pp65 fragment 1_F | GCCACCATGGAATTCATGGAGTCGCGCGGTCGCCG | 35 |
| pp65 fragment 1_R | ACTTCCTCTGCCCTCGCGGTGGCATGGCGTCGAGT | 35 |
| pp65 fragment 2_F | GCCACCATGGAATTCATCCTGGTGTCGCAGTACAC | 35 |
| pp65 fragment 2_R | ACTTCCTCTGCCCTCGCTGGGGATGTTCAGCATCT | 35 |
| pp65 fragment 3_F | GCCACCATGGAATTCATCTATGTGTACGCGCTGCC | 35 |
| pp65 fragment 3_R | ACTTCCTCTGCCCTCGACGTCGGGCTCTTTCCACT | 35 |
| pp65 fragment 4_F | GCCACCATGGAATTCCTGGCCTGGACGCGTCAGCA | 35 |
| pp65 fragment 4_R | ACTTCCTCTGCCCTCCACGTCCTCGCAGAAGGACT | 35 |
| pp65 fragment 5_F | GCCACCATGGAATTCGACCAGTACGTCAAGGTGTA | 35 |
| pp65 fragment 5_R | ACTTCCTCTGCCCTCGATGTGCGAGATCTTGCCCG | 35 |
| pp65 fragment 6_F | GCCACCATGGAATTCTGTCCCAAAAATATGATAAT | 35 |
| pp65 fragment 6_R | ACTTCCTCTGCCCTCCACGGGATCGTACTGACGCA | 35 |
| pp65 fragment 7_F | GCCACCATGGAATTCCAAGCGATACGCGAGACCGT | 35 |
| pp65 fragment 7_R | ACTTCCTCTGCCCTCCCAGACGTCGTCGTCGCCCT | 35 |
| pp65 fragment 8_F | GCCACCATGGAATTCGACCGGCACGACGAGGGTGC | 35 |
| pp65 fragment 8_R | ACTTCCTCTGCCCTCGCGGCCGCGTGTCATAACGC | 35 |
| pp65 fragment 9_F | GCCACCATGGAATTCTCCTCGGCGACGGCGTGCAC | 35 |
| pp65 fragment 9_R | ACTTCCTCTGCCCTCGTACTTCAGATTCTGACCCT | 35 |
| pp65 fragment 10_F | GCCACCATGGAATTCCTGGCCCGCAACCTGGTGCC | 35 |
| pp65 fragment 10_R | ACTTCCTCTGCCCTCACCTCGGTGCTTTTTGGGCG | 35 |
| T7-Kozak-F | TAATACGACTCACTATAGGGAGACCCAAGCTTGCCACCATGGAATTC | 47 |

| | | |
|---|---|---|
| Frag5-RPH mini-gene_F | GCCACCATGGAATTCTTCATGCGCCCCCACGAGCGCAACGGCTTTAC-GGTGTTGTGTCCCGAGGGCAGAGGAAGT | 75 |
| Frag5-VPS mini-gene_F | GCCACCATGGAATTCGAGGACGTGCCCTCCGGCAAGCTCTTTATGCAC-GTCACGCTGGGCGAGGGCAGAGGAAGT | 75 |
| Frag6-LMN mini-gene_F | GCCACCATGGAATTCGGTAACCTGTTGATGAACGGGCAG-CAAATCTTCCTGGAGGTACAAGAGGGCAGAGGAAGT | 75 |
| Frag8-TPR mini-gene_F | GCCACCATGGAATTCGAGCGTAAGACGCCCCGCGTCAC-CGGCGGCGGCGCCATGGCGAGCGAGGGCAGAGGAAGT | 75 |
| Frag9-NLV mini-gene_F | GCCACCATGGAATTCCTGGCCCGCAACCTGGTGCCCATGGTGGCTAC-GGTTCAGGGTCAGGAGGGCAGAGGAAGT | 75 |
| Frag10-RIF mini-gene_F | GCCACCATGGAATTCGACATCTACCGCATCTTCGCCGAATT-GGAAGGCGTATGGCAGCCCGAGGGCAGAGGAAGT | 75 |
| HIV-GPG mini-gene_F | GCCACCATGGAATTCGGAGTAGGAGGACCCGGCCATAAGGCAAGAG-TTTTGGCTGAAGCAGAGGGCAGAGGAAGT | 75 |
| HIV-FLG mini-gene_F | GCCACCATGGAATTCCAGGCTAATTTTTTAGGGAAGATCTGGCCTTCC-TACAAGGGAAGGGAGGGCAGAGGAAGT | 75 |
